# Feasibility of constant dose rate VMAT in the treatment of nasopharyngeal cancer patients

**DOI:** 10.1186/s13014-014-0235-1

**Published:** 2014-11-04

**Authors:** Wenliang Yu, Haijiao Shang, Congying Xie, Ce Han, Jinling Yi, Yongqiang Zhou, Xiance Jin

**Affiliations:** Radiation Oncology Department, Quzhou People’s Hospital, No. 2 Zhongloudi Street, Quzhou, 324000 China; Research department of IPTA(Beijing) Investment Administration Co., Ltd, Beijing, China; Department of Radiotherapy and Chemotherapy, the 1st Affiliated Hospital of Wenzhou Medical University, Wenzhou, 325000 China

**Keywords:** Volumetric-modulated arc therapy, Constant dose rate, Variable dose rate, Multicriteria optimization, Nasopharyngeal cancer

## Abstract

**Purpose:**

To investigate the feasibility of constant dose rate volumetric modulated arc therapy (CDR-VMAT) in the treatment of nasopharyngeal cancer (NPC) patients and to introduce rotational arc radiotherapy for linacs incapable of dose rate variation.

**Materials and methods:**

Twelve NPC patients with various stages treated previously using variable dose rate (VDR) VMAT were enrolled in this study. CDR-VMAT, VDR-VMAT and mutlicriteria optimization (MCO) VMAT plans were generated for each patient on RayStation treatment planning system with identical objective functions and the dosimetric differences among these three planning schemes were evaluated and compared. Non dosimetric parameters of optimization time, delivery time and delivery accuracy were also evaluated.

**Results:**

The planning target volume of clinical target volume (PTV-CTV) coverage of CDR-VMAT was a bit inferior to those of VDR- and MCO-VMAT. The V93 (p = 0.01) and V95 (percent volume covered by isodose line) (p = 0.04) for CDR-VMAT, VDR-VMAT and MCO-VMAT were 98.74% ± 0.31%, 99.76% ± 0.16%, 99.38% ± 0.43%, and 98.40% ± 0.48%, 99.53% ± 0.28%, 99.07% ± 0.52%, respectively. However, the CDR-VMAT showed a better dose homogeneity index (HI) (p = 0.01) in PTV-CTV. No significant difference in other target coverage parameters was observed. There was no significant difference in OAR sparing among these three planning schemes except for a higher maximum dose (Dmax) on the brainstem for CDR-VMAT. The brainstem Dmax of CDR-VMAT, VDR-VMAT and MCO-VMAT were 54.26 ± 3.21 Gy, 52.19 ± 1.65 Gy, and 52.79 ± 4.77 Gy, respectively. The average delivery time (p < 0.01) and the average percent γ passing rates (p = 0.02) of CDR-VMAT, VDR-VMAT and MCO-VMAT were 7.01 ± 0.43 min, 4.75 ± 0.07 min, 4.01 ± 0.28 min, and 95.75% ± 2.57%, 97.65% ± 1.45%, 97.36% ± 2.45%, respectively.

**Conclusion:**

CDR-VMAT offers an additional option of rotational arc radiotherapy for linacs incapable of dose rate variation with a lower initial cost. Its plan quality was acceptable but should be thoroughly checked compared with VDR-VMAT and MCO-VMAT in the treatment of NPC.

## Introduction

Due to its dose painting ability, intensity-modulated radiation therapy (IMRT), which is able to deliver more conformal radiation to the tumor than earlier techniques and spare the surrounding normal tissue from unnecessary exposure [1], has been accepted as a standard treatment modality for nasopharyngeal cancer (NPC) [2]. Disadvantages of IMRT are the longer required irradiation time and the greater total number of monitor units (MUs) compared with conventional methods [1]. The increased irradiation time may increase the intrafractional error and the patient restriction time. Additionally, an increase in the total number of MUs may expose the patients to the risk of low-dose irradiation owing to increased leakage [3].

More recently, volumetric-modulated arc therapy (VMAT), an extended form of IMRT using a variable dose rate (VDR), dynamic gantry speed and multileaf collimator (MLC) field shape, has gained considerable attention [4]. VMAT plans with shorter delivery time, fewer MU, and superior or equivalent plan quality compared to conventional step-and-shoot IMRT have been reported for prostate cancer and NPC [5,6]. However, to use the VMAT approach, the hardware and software systems of the radiation treatment planning system (TPS), as well as the linear accelerator (linac), must be upgraded. These upgrades are costly. Thus, many institutions do not have the means to perform VMAT and must continue with the problems specified above. Constant dose rate (CDR) VMAT is a rotational IMRT technique that uses a fixed gantry rotational speed and a fixed dose rate. Compared with fixed field IMRT, the potential advantages of CDR with a great reduction in irradiation time and clinical acceptable dose distribution has been reported [5,7]. For institutions that do not currently perform VDR-VMAT, CDR-VMAT may be a useful option.

Multicriteria optimization (MCO) with multipareto fronts has emerged as a tool for comparing treatment plans as it gives better visualization of the trade-offs [8-10]. MCO allows trade-offs between conflicting objectives encountered in VMAT planning to be explored in an interactive manner through search over a continuous representation of the relevant treatment options. MCO-VMAT plans in the treatment of prostate, pancreas, lung, and head and neck cancer patients have been reported with comparable dose distribution quality to conventionally optimized VMAT plans [11]. During the MCO optimization process, despite protocols, the final choice of the best solution will be planner dependent. However, in comparing Pareto fronts for different techniques, a bias towards planner experience can be minimized.

NPC is the most common H&N malignancy in Southeast Asia and China. The newly VDR-VMAT is an ideal treatment technique in NPC treatment but cost too much in linac upgrading. The purpose of this study is to investigate the dosimetric differences among CDR-VMAT, VDR-VMAT and MCO-VMAT and the feasibility of CDR-VMAT in the treatment of NPC patients.

## Materials and methods

### Patients and contours

Twelve patients with various stages of NPC treated previously by VDR-VMAT were enrolled in this study. All VDR-VMAT plans with initial CT data were exported from the Pinnacle TPS to the Raystation TPS (clinical version 3.5, RaySearch Laboratories, Stockholm, Sweden) through the DICOM service and the dose distributions were recalcuated based on the same CT number to density calibration curve using a collapsed cone dose engine with a dose grid of 4 mm × 4 mm × 4 mm. CDR-VMAT, VDR-VMAT and MCO-VMAT plans were generated for each patient on the RayStation TPS. The Raystation TPS was commissioned with the same beam data as the Pinnacle system. The dose deviations between Raystation and Pinnacle were within 1.5% during the commission process. Dosimetric differences among these three planning methods were compared.

**Figure 1:**
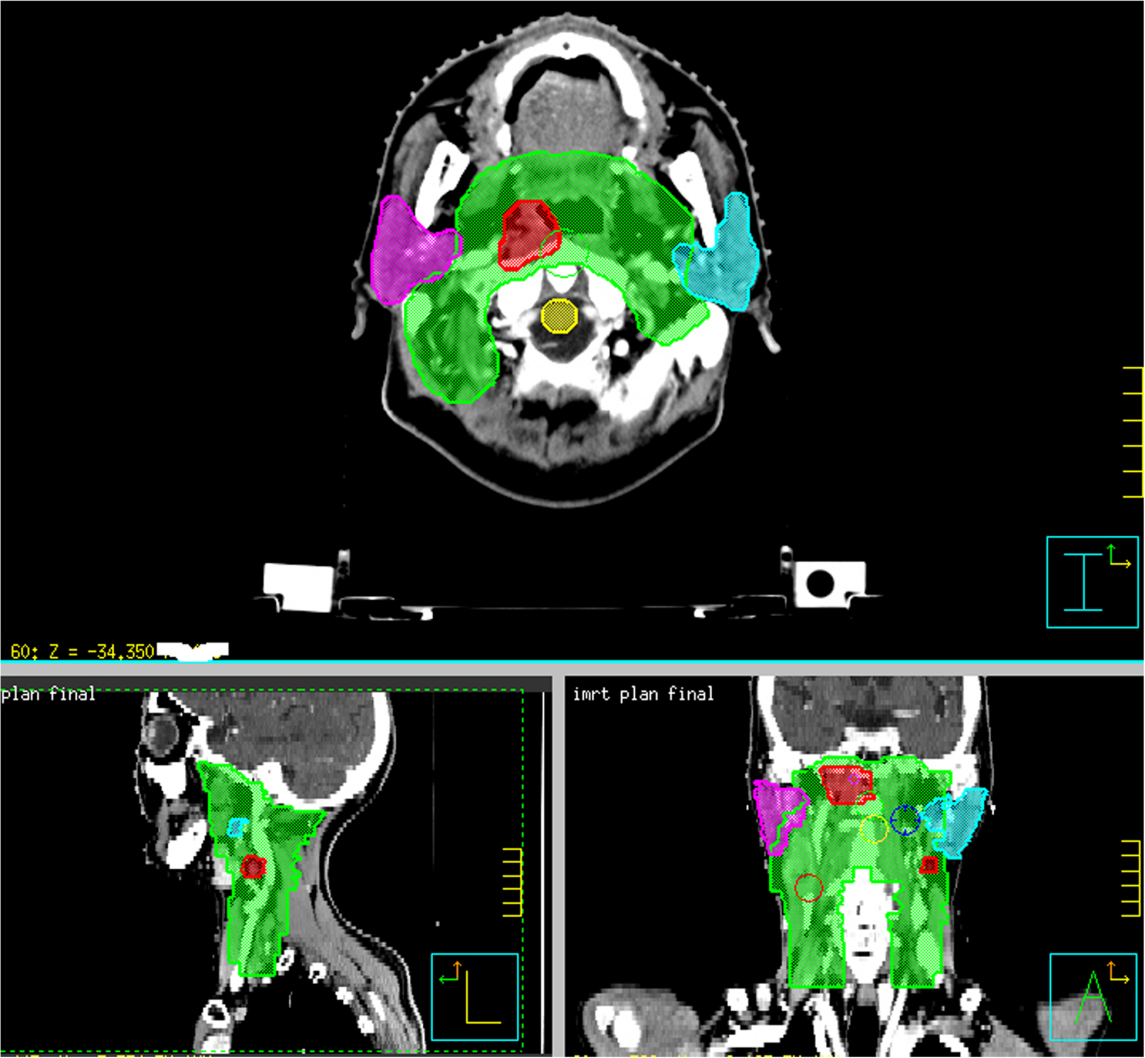
**Typical target contours for nasopharyngeal cancer patients, green for CTV, red for GTV, pink for right parotid, sky blue for left parotid, and yellow for spinal cord.**

Target and normal tissue contours have been reported in our previous study and are generalized here [12]. Gross tumor volume (GTV) was delineated as the mass shown in the enhanced computed tomography images or magnetic resonance images or both. The clinical target volume (CTV) was defined as the GTV plus a margin of potential microscopic spread. One typical contour is shown in Figure 1. The planning target volume (PTV) was created by adding a 3 mm margin to the CTV account for setup variability. In order to increase the coverage of the GTV, a PTV-GTV was generated by adding a 2 mm margin to the GTV. Organ at risks (OARs) (brain stem, spinal cord, lens, left and right parotids) were contoured and constrained for optimization.

### Treatment planning

The prescription doses to PTV-GTV and PTV-CTV were 2.5 Gy and 2.0 Gy per fraction for a total dose of 70 Gy and 56 Gy in 28 fractions with simultaneous integrated boost (SIB), respectively. VDR-VMAT objective settings were employed in the optimization for all plans as shown in Table 1. The optimization parameters of VDR-VMAT have been reported in our previous study and are summarized briefly here [6]. Leaf motion constraints of 0.46 cm/deg and a final arc spacing of 4 degree were employed. A start angle of 181 degree and a stop angle of 180 degree were applied for the first arc in a clockwise direction. The second arc rotated counterclockwise from 181 to 180 degree. For CDR-VMAT plans, a fixed dose rate of 400 MU/minute was selected. The collimator was set to 45 degree for all VMAT plans to reduce the cumulative effects of interleaf transmission and the tongue and groove effect. Other settings (like leaf motion, beam numbers and beam rotation) of CDR-VMAT were the same as the VDR-VMAT.

**Table 1:** **Objective setting and weight for inverse optimization**

**ROI**	**Type**	**Target(cGy)**	**% Volume**	**Weight**
PTV-GTV	Min Dose	6930		20
PTV-GTV	Uniform Dose	7000		10
PTV-GTV	Max Dose	7700		10
PTV-CTV	Min Dose	5544		20
PTV-CTV	Uniform Dose	5600		10
PTV-CTV	Max Dose	6160		10
Brainstem	Max Dose	5000		1
Cord	Max Dose	4000		1
R parotid	Max EUD A1	2600	40	1
L parotid	Max EUD A1	2600	40	1
lens	Max Dose	700		1
1 cm Ring out CTV	Max DVH	5600	10	1

For MCO optimization, the Pareto surface was approximated by using N +1 treatment plans, where N is the number of objectives. N of the plans are from each of the objectives being minimized individually. The additional plan is a balanced plan formed by optimizing the equal weighted sum of all objectives. A senior physician will navigating the base plans and decided the best plan. When navigation is completed, the Raystation TPS will create a deliverable version of the selected plan by a direct aperture optimization algorithm using a total of 50 segments as VDR-VMAT and CDR-VMAT to match the navigated DVH.

### Plan evaluation and comparison

The dose distributions delivered by the three modalities were compared by evaluating target coverage (TC) and OAR sparing. The maximum dose (Dmax), minimum dose (Dmin), mean dose (Dmean), the volumes receiving 93% (V93) and 95% (V95) of the prescribed dose of PTV-GTV and PTV-CTV; the Dmax of brainstem, spinal cord, lens, and the Dmean of parotids were evaluated and compared. For parotids, the percent volume of each parotid that received 26 Gy (V26), and 32 Gy (V32) was also evaluated and compared. Additional quality indices were calculated for further evaluation and comparison as listed in the following paragraphs.

The homogeneity index (HI) was evaluated as the difference between the dose to 1% (D1) and 99% (D99) of PTV divided by the prescription dose (Dp) [13]

1 $$ HI=\frac{D1-D99}{Dp}\times 100\% $$

Conformity index (CI) [14] and conformation number (CN) [15] were also calculated for the PTV as

2 $$ \mathrm{C}\mathrm{I}=\frac{{\mathrm{V}}_{\mathrm{T},\kern0.5em \mathrm{Pi}}}{{\mathrm{V}}_{\mathrm{Pi}}} $$

3 $$ \mathrm{C}\mathrm{N}=\frac{{\mathrm{V}}_{\mathrm{T},\mathrm{Pi}}}{{\mathrm{V}}_{\mathrm{T}}}\times \frac{{\mathrm{V}}_{\mathrm{T},\mathrm{Pi}}}{{\mathrm{V}}_{\mathrm{Pi}}} $$

Where V_T,Pi_ is the volume of the target that is covered by the prescription dose, V_T_ is the target volume. V_Pi_ is the volume of the body that is covered by the prescription isodose. The maximum value of the CI is 1, corresponding to a perfect coverage of the PTV. The CN is the complementary information to compensate for the defects of target coverage and CI. The CN can take values between 0 and 1, where an ideal dose distribution would have a CN value of 1.

The VMAT plans were verified by an ArcCheck phantom and the SNCP Patient analysis software (Version 6, Sun Nuclear Corporation, Melbourne, FL) with a gamma criterion of 3%/ 3 mm. For profile analysis, a threshold dose of 10% of the maximum dose was used. All plans were delivered on an Elekta Synergy linac (Elekta Ltd., Crawley, UK) with a MOSAIQ record and verify system (version 1.60Q3, IMPAC Medical Systems, Inc.,Sunnyvale, CA). Besides, the optimization time, MU efficiency, and delivery time were compared.

### Statistical analysis

The differences among the three planning schemes were analyzed using one-way ANOVA. When an overall significant difference was observed, the post hoc Turkey’s test was used to determine which pair-wise comparisons differed. All statistical analyses were conducted with the SPSS 17.0 software. Differences were considered statistically significant for p <0.05.

## Results

A total of 36 VMAT plans were generated for 12 NPC patients (3 female and 9 male) with an average age of 58.5 years (48–71). Table 2 lists the clinical characteristics of these 12 patients. The average PTV-GTV and PTV-CTV volumes were 82.1 cm^3^ (59.6-171.0) and 594.1 cm^3^ (416.5-757.7), respectively.

**Table 2:** **Patients characteristics**

**Patients no.**	**Staging**	**Gender**	**Age**	**PTC-CTV volume (cm** ^**3**^ **)**	**PTV-GTV volume (cm3)**
1	T2N2MX	M	48	618.0	59.6
2	T2N1MX	M	63	757.7	85.5
3	T3N0M0	M	71	589.1	62.2
4	T4N0MX	M	59	546.8	171.0
5	T3N3MX	M	71	504.2	92.4
6	T2N2MX	F	66	416.5	64.4
7	T4N0MX	M	55	739.7	83.4
8	T2N2MX	M	39	498.1	45.3
9	T2N3MX	M	48	462.4	45.8
10	T1N1M0	M	62	603.8	93.1
11	T2bN1M0	F	55	725.6	89.7
12	T2bN2M0	F	65	668.0	92.8

**Figure 2:**
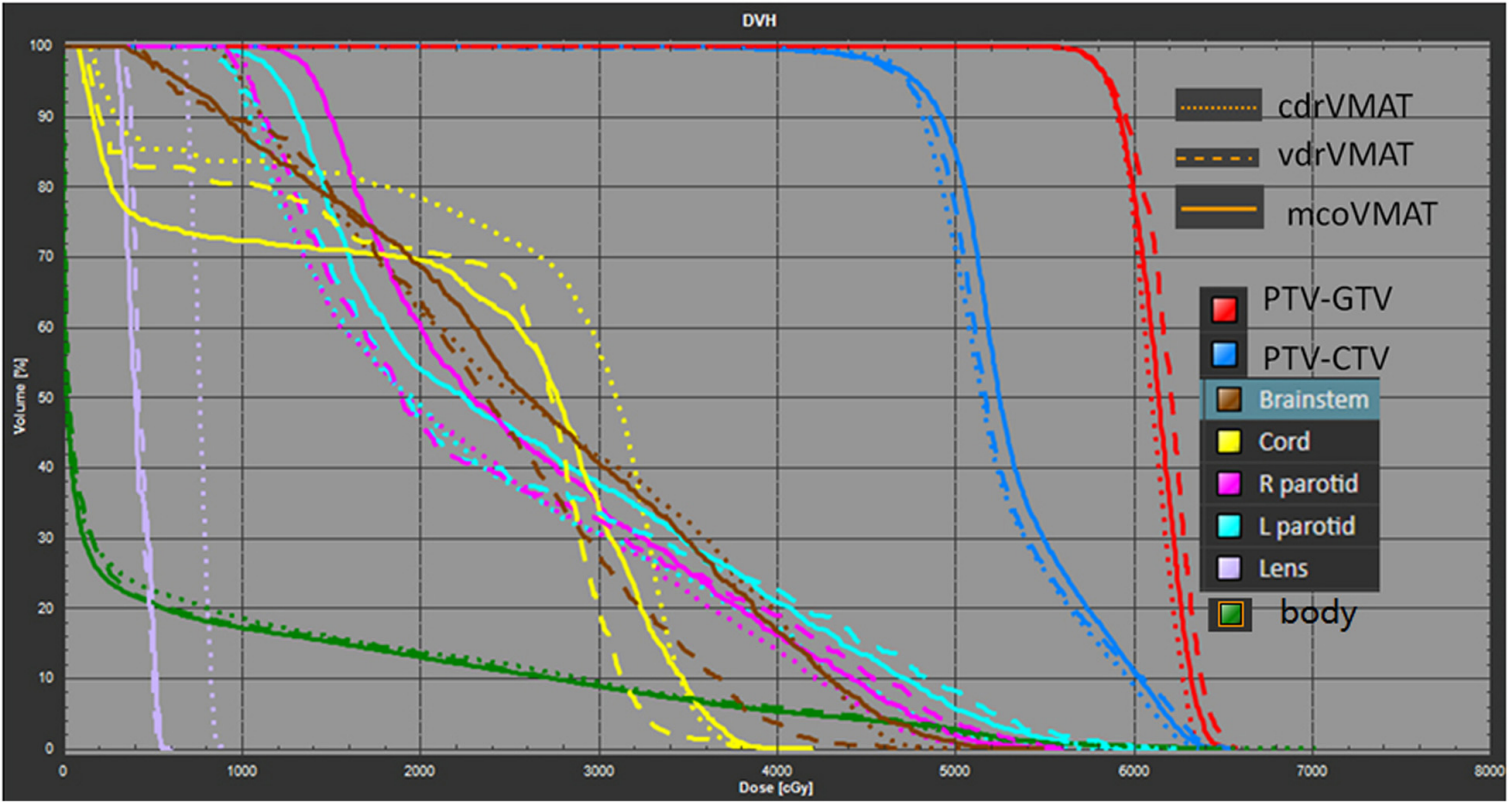
**DVH comparison among CDR-VMAT, VDR-VMAT and MCO-VMAT for one NPC patient.**

Figure 2 shows a typical DVH comparison for one patient among these three planning schemes. For this particular patient, a similar PTV-GTV coverage is observed with less hotspot in CDR-VMAT. The PTV-CTV coverage of MCO-VMAT was better as compared with the other two planning schemes. VDR-VMAT presented a better sparing of the spinal cord and brainstem and CDR-VMAT showed the worst lens protection compared with the other two schemes. Table 3 presents the detailed statistical analysis of target coverage for all the patients. No significant difference in PTV-GTV coverage was shown. The V93 (p = 0.01) and V95 (p = 0.04) of PTV-CTV for CDR-VMAT, VDR-VMAT and MCO-VMAT were 98.74% ± 0.31%, 99.76% ± 0.16%, 99.38% ± 0.43%, and 98.40% ± 0.48%, 99.53% ± 0.28%, 99.07% ± 0.52%, respectively. The HIs for CDR-VMAT, VDR-VMAT and MCO-VMAT were 0.28 ± 0.017,0.29 ± 0.012, 0.31 ± 0.023, respectively (p = 0.01). CDR-VMAT showed a poorer PTV-CTV coverage but with a better dose homogeneity index compared with the other two planning schemes. No significant difference in other target coverage parameters was observed.

**Table 3:** **Target coverage comparison**

	**CDR-VMAT**	**VDR-VMAT**	**MCO-VMAT**	**p**
PTV-GTV				
Dmean (Gy)	68.41 ± 1.21	68.71 ± 1.28	68.34 ± 0.47	0.42
Dmax (Gy)	71.02 ± 0.93	70.66 ± 1.29	70.44 ± 0.63	0.55
V93 (%)	99.91 ± 0.18	99.93 ± 0.04	99.98 ± 0.02	0.31
V95 (%)	99.72 ± 0.38	99.69 ± 0.13	99.83 ± 0.16	0.38
HI	0.11 ± 0.021	0.12 ± 0.016	0.11 ± 0.026	0.33
CI	0.62 ± 0.64	0.57 ± 0.10	0.65 ± 0.06	0.43
CN	0.62 ± 0.44	0.56 ± 0.11	0.63 ± 0.05	0.26
PTV-CTV				
Dmean (Gy)	56.65 ± 0.47	57.50 ± 1.08	56.15 ± 0.62	1.13
Dmax (Gy)	70.09 ± 0.83	70.76 ± 1.06	70.57 ± 0.43	0.62
V93 (%)	98.74 ± 0.31	99.76 ± 0.16	99.38 ± 0.43	0.01
V95 (%)	98.40 ± 0.48	99.53 ± 0.28	99.07 ± 0.52	0.04
HI	0.28 ± 0.017	0.29 ± 0.012	0.31 ± 0.023	0.01
CI	0.58 ± 0.14	0.57 ± 0.10	0.60 ± 0.06	0.06
CN	0.58 ± 0.04	0.56 ± 0.12	0.57 ± 0.15	0.12

The comparison of OAR sparing is presented in Table 4. The average Dmax of the brainstem for CDR-VMAT, VDR-VMAT and MCO-VMAT was 54.26 ± 3.21 Gy, 52.19 ± 1.65 Gy, and52.79 ± 4.77 Gy, respectively (p = 0.01). The average Dmax of the brainstem of CDR-VMAT was higher (p = 0.02) than the other two methods. No significant difference in other OAR parameters was observed among these three planning schemes. For non dosimetric parameter comparisons, the average MU values of CDR-VMAT, VDR-VMAT and MCO-VMAT were 544.88 ± 106.95, 565.88 ± 25.34, 526.38 ± 54.38, respectively (p = 0.06). The average delivery times of CDR-VMAT, VDR-VMAT and MCO-VMAT were 7.01 ± 0.43 min, 4.75 ± 0.07 min, 4.01 ± 0.28 min, respectively (p < 0.01). The average percent γpassing rates were 95.75% ± 2.57%, 97.65% ± 1.45%, and 97.36% ± 2.45%, respectively (p = 0.02). The average optimization time of CDR-VMAT (24.74 ± 1.06 min) was shorter than VDR-VMAT (40.8 ± 4.83 min). The optimization time of MCO-VMAT was short (5.36 ± 2.55 min), but it took a long and variable navigation time, which is about 40 minutes.

**Table 4:** **OAR sparing comparison**

	**CDR-VMAT**	**VDR-VMAT**	**MCO-VMAT**	**p**
Brainstem				
Dmax (Gy)	54.26 ± 3.21	52.19 ± 1.65	52.79 ± 4.77	0.01
Cord				
Dmax(Gy)	45.12 ± 4.94	44.59 ± 2.10	44.11 ± 4.37	0.47
Left parotid				
Dmean (Gy)	29.72 ± 1.48	28.42 ± 1.75	28.46 ± 2.70	0.93
V26 (%)	53.84 ± 4.41	50.55 ± 3.75	49.73 ± 8.82	0.34
V32 (%)	34.36 ± 5.50	33.22 ± 5.28	33.01 ± 9.33	0.16
V50 (%)	6.44 ± 5.05	6.55 ± 5.65	5.72 ± 4.58	0.68
Right parotid				
Dmean (Gy)	29.79 ± 1.73	28.76 ± 1.15	28.6 ± 2.18	0.98
V26 (%)	48.04 ± 3.90	47.38 ± 3.75	48.45 ± 7.99	0.42
V32 (%)	31.78 ± 5.80	30.22 ± 5.35	30.84 ± 8.28	0.30
V50 (%)	7.07 ± 3.81	6.17 ± 3.93	6.26 ± 3.34	0.45

## Discussion

This study has investigated the dosimetric differences among CDR-VMAT, VDR-VMAT and MCO-VMAT in the treatment of NPC patients and the feasibility of CDR-VMAT. Dual arc CDR-VMAT is able to achieve acceptable target coverage and OAR sparing compared with VDR-VMAT and MCO-VMAT in the treatment of NPC patients.

Due to the limited availability of linacs with reliable variable dose rate capability, CDR-VMAT has been investigated in order to deliver rotational arcs using a constant dose-rate with a lower initial cost compared with VDR-VMAT [16]. In this study, CDR-VMAT achieves a similar target coverage, dose homogeneity and conformity of the PTV-GTV compared with VDR-VMAT and MCO-VMAT. However, CDR-VMAT was a bit inferior on the coverage on PTV-CTV and superior in dose homogeneity. Similar results have been reported, showing that the differences in the GTV and PTV coverage for head-and-neck cancer patients (dose received by 95% of GTV or PTV) in the VDR- and CDR-VMAT plans were within 0.1% [17]. The maximum dose to the brainstem for CDR-VMAT was a bit higher compared with the other two planning schemes, but it was still within the clinical tolerance. There was no other significant difference on OAR sparing among these three VMAT planning schemes.

MCO with a pareto surface-based technique has been demonstrated as a method to efficiently explore tradeoffs between different treatment goals in the irradiation of various tumors. Treatment plans selected from such a representation are of comparable dose distribution quality to conventionally optimized VMAT plans [11]. In this study, consistent results were observed with equivalent dose distribution for MCO-VMAT and VDR-VMAT in the treatment of NPC. The equivalent dosimetric distribution of CDR-VAMT was further verified by MCO-VMAT in this study.

The delivery efficiency and MU reduction are some of the features that VMAT was favored over conventional fixed field IMRT. The average delivery time of CDR-VMAT was longer than VDR-VMAT (7.01 ± 0.43 min vs. 4.75 ± 0.07 min), but it was still much shorter than that of IMRT (11.01 ± 0.43 min) as reported in our previous study [6]. All the CDR-VMAT plans were delivered smoothly and accurately in this study with an average percent γpassing rate of 95.75% ± 2.57%. This is consistent with the study of Tang et al. [17]

VDR-VMAT both with single arc and dual arc has been widely investigated in the treatment of various tumor sites with superior or equivalent dose distribution and reduced delivery time and MUs [5,6,18]. However, the VDR-VMAT capability is not available for the low- or intermediate-end linacs without upgrade in institutions especially in the underdeveloped countries and regions. A careful study in the dosimetric distribution and feasibility of CDR-VMAT in such institutions will be of clinical benefit for both hospitals and patients.

## Conclusion

CDR-VMAT offers an additional option of rotational arc radiotherapy for linacs incapable of dose rate variation with a lower initial cost. Its plan quality is acceptable but should be checked with care compared to VDR-VMAT and MCO-VMAT in the treatment NPC.
